# The Protective Effect of Decentering on the Links Between Internalising Symptoms, Functional Impairment and Wellbeing

**DOI:** 10.1016/j.beth.2026.04.002

**Published:** 2026-04-08

**Authors:** Tanika R. Sgherza, Rodrigo Becerra, Lauren M. Bylsma, David M. Fresco, Kristin Naragon-Gainey

**Affiliations:** University of Western Australia; University of Western Australia; University of Pittsburgh; University of Michigan; University of Western Australia

**Keywords:** Decentering, functional impairment, well-being, anxiety, depression

## Abstract

There is a lack of longitudinal research examining the relationships between internalising symptoms and well-being via functional impairment. *Decentering* is a malleable and clinically relevant characteristic that may disrupt the links among these constructs over time, but this is yet to be explored. A community sample (*N* = 386) completed monthly self-report surveys for 12 months, assessing internalising symptoms (depression, generalised anxiety, social anxiety, panic), impairment, well-being and decentering. Multilevel structural equation modelling was conducted to analyse the within-person association between symptoms and well-being mediated by impairment (in the same month and subsequent months), as well as a moderated-mediation model with decentering as the moderator (in the same month and as individual differences). Further, the mediation model was tested over time bidirectionally. Within a given month, higher levels of symptoms significantly predicted lower well-being via impairment, and prospective analyses revealed that these effects were bidirectional. Decentering significantly reduced the impact of one’s symptoms on impairment on a given month and throughout the study, but not the impact of impairment on well-being. Thus, decentering was supported as a protective factor that reduces the link between symptoms and impairment. Further research is needed to uncover different ways to protect well-being, particularly because low well-being also predicted elevated symptoms over time. These novel findings indicate that the inverse cycle between symptoms and well-being may be maintained by impairment, but the short-term and cumulative effects of symptoms on impairment could be reduced by decentering. As such, impairment and decentering may be important therapeutic targets when aiming to promote well-being in the context of symptoms.

Good psychosocial functioning and well-being are important clinical targets that are often negatively impacted by internalising symptoms (i.e., anxiety and depression, referred to as “*symptoms*” throughout this paper). Theory suggests that when functioning in major life domains (e.g., work, school, daily tasks, home life) becomes impaired due to symptoms (i.e., *functional impairment*), this reduces *well-being* (i.e., life satisfaction, sense of meaning and purpose, happiness; Keyes, 2005; Huppert, 2009). Empirical studies have tested this in mediation models, finding that symptoms elicit detrimental effects on well-being (and relatedly, quality of life) indirectly, via impairments in functioning (Sgherza et al., 2025; Zhou et al., 2022).

Although internalising symptoms are often linked with impaired functioning and diminished well-being, these associations show substantial heterogeneity across individuals (Kiel et al., 2024; McKnight & Kashdan, 2009; McKnight et al., 2016; Rodríguez et al., 2012). Some people with significant symptoms maintain relatively intact functioning and experience high levels of well-being (Magalhães, 2024; Orpana et al., 2016; Zimmerman et al., 2006), suggesting that the presence of symptoms alone does not determine outcomes. For example, a study by Orpana et al. (2016) found that one third of participants with mood and anxiety disorders reported high well-being, and this was associated with fewer functional impairments. This variability highlights the need to examine regulatory and contextual moderators that shape how symptoms are managed and translated to these outcomes. Identifying such modifiable mechanisms is essential for understanding who maintains adaptive functioning and well-being despite symptoms—and why.

## protective effect of decentering

One potentially malleable and clinically-relevant characteristic that has not yet been explored in the context of associations among internalizing symptoms, functional impairment, and well-being is *decentering* (Bernstein et al., 2015; Fresco et al., 2007; Teasdale et al., 2000), Decentering is conceptualised as the capacity to observe thoughts and feelings as temporary, objective mental events rather than facts or enduring traits that reflect the self (Fresco et al., 2007; Safran & Segal, 1990). Decentering-related abilities are relevant to reducing transdiagnostic mechanisms of psychopathology (e.g., rumination, negative cognitive appraisals, Panagou & MacBeth, 2022; Wu et al., 2022) and are a core component through which interventions promote resilience and positive mental health outcomes (Bennett et al., 2021).

There are several mechanisms through which decentering may interrupt the progression from symptoms to impairment and diminished well-being. For example, King and Fresco (2019) state that decentering promotes emotional recovery by fostering flexible engagement with distressing experiences; it also facilitates adaptive repetitive thought (e.g., savouring gratitude), which can counteract maladaptive repetitive thought (Lawrence et al., 2021), and promotes greater coping efficacy and flexibility (Duncan et al., 2021) and reduced reactivity (Milosch, 2019). In the context of impairment, individuals who distance themselves from pessimistic appraisals may sustain goal-directed behaviour and preserve functioning even during periods of symptom elevation. Likewise, decentering may buffer the impact of functional setbacks by supporting more adaptive interpretations or reappraisals, thereby protecting well-being. This notion is empirically supported through observational, clinical trials and longitudinal research (Wu et al., 2022; Stange et al., 2017; Johannsen et al., 2022; Moore et al., 2022). Thus, decentering appears to potentially disrupt links between symptoms, impairment, and reduced well-being.

It is plausible that decentering could interrupt several paths in the association from symptoms to impairment and poor well-being. First, decentering may prevent symptoms from impairing functioning. Research suggests that high functioning individuals (i.e., high well-being and less impairment during or after psychopathology) effectively regulate emotions and are psychologically flexible, which could be facilitated by processes such as decentering (Rottenberg et al., 2018). Kaiser et al. (2015) postulated that appraisals of and responses to emotional experiences may shape the effect of the experience on behavioural functioning. For example, responding to problems with overwhelm and pessimism is a strong predictor of impaired work functioning for people with major depressive disorder (De Vries et al., 2015). Decentering may help to reduce such feelings and increase capacity to cope with problems by becoming less immersed in negative emotions and thoughts (Duncan et al., 2021; Milosch, 2019; Naragon-Gainey et al., 2023). Moreover, for people experiencing high distress, greater decentering promotes goal pursuit, as it increases approach motivation (Eftekhari et al. 2017). Such motivation is key to maintaining or thriving in functioning domains such as work/study, hobbies, and day-to-day tasks.

Second, when functioning does become impaired or difficult, individuals may experience increased stressors (e.g., poor work evaluation, relationship difficulties, losing things due to disorganisation), as well as demoralisation, distress, and shame, which could have flow-on effects on well-being (Chao, 2014). However, decentering can facilitate adaptive coping with these stressors (e.g., interpersonal stress, Duncan et al., 2021) and the associated negative emotions (Fuochi & Voci, 2024), serving to preserve well-being. Consistent with these findings, the Mindfulness-to-Meaning Theory suggests that decentering in the face of such adverse experiences promotes positive reappraisal, positive affect, savouring, elicits a sense of meaning, and dampens the intensity and persistence of distress that may be associated with these stressors (Bennett et al., 2021, Garland et al., 2015; Naragon-Gainey et al., 2023; Sgherza et al., 2022). Taken together, there is reason to believe that decentering may be protective against (a) the effects of symptoms on impairment, and (b) the effects of impairment on well-being.

## dynamic associations over time

Understanding how symptoms, impairment, and well-being relate within individuals over time is essential for modelling real-world emotional dynamics and therapeutic change. Although much of the existing literature is cross-sectional, emerging research has begun to characterize these constructs as temporally variable. Studies using daily or momentary assessments have identified inverse associations between symptoms and well-being, revealing meaningful fluctuations across days and contexts (Kraiss et al., 2022; Mason-Stephens et al., 2025). Impairment appears to vary as well, with evidence that elevations in depressive symptoms correspond to concurrent increases in functional disruption over the course of a month (Judd et al., 2000). Decentering, too, shows within-person variability, with levels shifting across situations and over time (Goodman et al., 2018; Stange et al., 2017; Wu et al., 2022). More specifically, a study by Naragon-Gainey et al. (2023) showed that momentary decentering moderated the affect-well-being relationship at the state level, beyond trait relationships, suggesting momentary buffering effects at the within-person level. Similarly, Forchuk et al. (2025) also found that momentary decentering reduces the relationship between stress and affect. More generally, concurrent momentary associations between state defusion (a related construct) and affect have been observed in other daily life studies (e.g., Krafft et al., 2021). Clarifying how these time-varying processes unfold—especially whether greater decentering moderates or attenuates the effects of symptom escalation on functioning and well-being—could help illuminate when and for whom decentering acts as a protective mechanism.

The relationship between symptoms and well-being mediated by impairment has not been tested longitudinally and prospectively, which is necessary for providing a strong test of potential causal relationships and directionality of effects (Maric et al., 2012). One possibility is that functional difficulties accumulate over time, such that persistent impairment eventually undermines well-being (e.g., sustained work strain leading to job loss; unresolved interpersonal stress culminating in relationship dissolution). Past longitudinal studies have shown that depressive symptoms influence subsequent impairment (Curtiss et al., 2018), which contributes to poorer quality of life (related to well-being) over years (Goldberg & Harrow, 2005). However, these processes have yet to be evaluated within an integrated, temporally sensitive model.

In regard to directionality, it is possible that bidirectional effects exist among these variables, though this has not yet been directly tested, leading to feedback loops wherein these networks of variables maintain or worsen symptoms and well-being over time (Lamers et al., 2015; Joshanloo & Blasco-Belled, 2023). While there is evidence to suggest that greater well-being is associated with lower risk of symptoms over time (Joshanloo & Blasco-Belled, 2023; Keyes et al., 2010; Lamers et al., 2015; Wood & Joseph, 2010), there is mixed evidence about whether aspects of well-being are associated with improvements in role functioning over time (Moskowitz et al., 2012). Thus, it is unclear whether well-being impacts later symptoms due to effects on functioning or via another mechanism. Testing alternative temporal models using longitudinal data is essential for clarifying directionality and identifying intervention targets that promote resilience and recovery.

## current study

Although it is known that impairment plays a mediating role in the relationship between symptoms and well-being, the ways in which these detrimental effects can be prevented is not understood. Decentering—a metacognitive skill that can be cultivated over time—has not been tested as a protective moderator within this network. Additionally, the temporal ordering among symptoms, impairment, and well-being remains unclear, as does the possibility of bidirectional effects. Thus, to address these gaps, the present study used a longitudinal design that assessed symptoms, impairment, well-being and decentering monthly for twelve months.

The aims of this study were twofold. Primarily, this study aimed to evaluate whether the mediation pathways among symptoms, impairment, and well-being are moderated by one’s tendency to decenter (relative to one’s norm) when examining fluctuations from month-to-month in these variables. We hypothesised at least partial mediation, such that the indirect effect from symptoms to well-being via impairment in a given month would be significant. Moreover, we hypothesised that in months where decentering is greater than one’s norm, the associations between (a) symptoms-impairment, and (b) impairment-well-being during that same month will be reduced. That is, decentering will have a protective effect on impairment and well-being. As an exploratory aim, this model was also investigated as individual differences (i.e., overall scores across 12 months), rather than fluctuations over time. This model tests whether people who tend to decenter more throughout the study period tend to have weaker associations among symptoms, impairment, and well-being.

A secondary aim was to test whether the components of the indirect pathway preceded one another temporally, as the mediation model would imply. Specifically, a lagged mediation model was run in which symptoms (T1), impairment (T2) and well-being (T3) were each temporally separated by one month to capture longer term associations and cumulative effects of impairment that may otherwise be missed in concurrent within-person models. Moreover, we assessed this mediation model in the reverse-order (with well-being at T1 and symptoms at T3) as an alternative model and to determine if there are bidirectional associations that indicate feedback loops whereby current well-being also impacts future functioning and symptoms. We tentatively hypothesised at least partial mediation for both models.

## Method

We conducted secondary data analysis on a larger dataset, and the research questions, measures, and analyses for the current study were pre-registered on Open Science Framework prior to conducting analyses: https://osf.io/skunq/?view_only=9b4e7336299642cd94ac67944f9efd66. No other published manuscripts from this dataset report this set of variables together.

### PARTICIPANTS

Community participants were recruited in Perth, Western Australia from 2020–2024 using advertisements on social media, job websites, flyers, university websites, and word of mouth. Participants completed an online screening questionnaire (and where necessary, follow-up phone interviews) and were excluded from the larger study due to the following reasons: younger than 18 or older than 65; current mania, psychosis or substance use disorder; pregnancy; cardiovascular condition or medication that impacted heart rate variability. Eligible participants were invited to enroll in Phase 1 (baseline phase, not analysed in the present study). After completion of Phase 1 and/or Phase 2 (ecological momentary assessment phase, not analysed in the present study), participants were invited to enroll in Phase 3 (follow-up phase). The follow-up phase consisted of brief online self-report surveys for 12 months.

The sample consisted of 386 participants who enrolled in the follow-up phase of the larger study. Participants were aged 18 to 65 (*M* = 34.49, *SD* = 13.56), mostly identified as female (60.9%), single (53.12%) and White (63.72%) or Asian (30.58%). Less than 1% (0.8%) of the sample identified as Aboriginal or Torres Strait Islander. Thirty-seven percent of the sample were full-time students and 7.3% were part-time students. Thirty-one percent were employed part-time, 18.9% were employed full-time, and 21.5% were unemployed (note multiple selections were possible for student and employment status). Over half (53.88%) of the participants had a history of psychological treatment at baseline. Approximately one quarter (22.8%) currently met criteria for Generalised Anxiety Disorder and 12.4% met criteria for unipolar depressive disorders at baseline.

Participant self-report data were excluded (4.3% of responses) if one or more inattention checks were failed (i.e., responding incorrectly to >1 attention check items, average responses to invalidity items >2 SDs from the sample mean, and/or speeded responding as defined by Greszki’s (2015) QualiTime index). Twelve participants did not complete the monthly follow-up phase (i.e., withdrew at some point). On average, people who completed the whole phase submitted 9.96 of 12 possible surveys (mean surveys completed = 83%, *SD* = 26%, Range = 8–100%). There was an average of 30.51 days between each completed survey (*SD* = 2.73, Range = 18.18–42.64 days).

### PROCEDURE

The follow-up phase commenced 1 month after Phase 1 completion. Surveys were emailed out monthly via Qualtrics and took 10–15 minutes to complete. Participants had one week to fill out the survey once it had been sent and were paid $10 AUD for each completed survey. As an extra incentive, participants were also entered into a raffle to win an iPad if they completed at least 8 of 12 surveys. This study was approved by the Human Research Ethics Committee at The University of Western Australia.

### MEASURES

#### Inventory of Depression and Anxiety Symptoms – Expanded Version (IDAS-II; Watson et al., 2012)

This 99-item instrument is designed to assess anxiety and depression. Participants rated how much they have felt or experienced each symptom in the past month, using a 5-point Likert scale from 1 (Not at all) to 5 (Extremely). The current study uses the Dysphoria (9 items), Anxious Mood (7 items), Social Anxiety (5 items) and Panic (8 items) scales. Higher scores indicate greater symptom severity. Note that there is one identical item in the Dysphoria and Anxious Mood scales, so it was removed from Dysphoria to eliminate item overlap across scales. The IDAS subscales demonstrated excellent reliability in the current study (Cronbach’s α = .87 to .95) comparable to prior studies (Cronbach’s α = .90 to .91 overall; Sanchez-Garcia et al., 2021; Weitzner et al., 2020).

#### World Health Organisation Disability Assessment Schedule 2.0 (WHODAS 2.0; Üstün et al., 2010)

The WHODAS is a 36-item measure of global functioning and disability, assessing six different domains: Cognition, Mobility, Self-Care, Getting Along, Life Activities and Participation. Participants were asked to rate how much difficulty they have had with each activity due to health conditions (including mental and physical conditions) over the past month, using a 5-point Likert scale from 1 (*None*) to 5 (*Extreme* or *Cannot Do*). Scores were summed for each subscale, with higher score reflecting greater functional impairment. The WHODAS demonstrated excellent reliability across all subscales (Cronbach’s α = .71 to .97). This is comparable to previous studies (Cronbach’s α = .91 to .97 overall; Higgins et al., 2021; Norén et al., 2023; Subramaniam et al., 2020). Discriminant validity for the WHODAS 2.0 has been established in a previous study, with significant but not excessively high associations with psychological distress (measured with Kessler Distress Scale, *r* = −.60) and with well-being (measured with Mental Health Continuum Short-Form, *r* = .43; Plouffe et al., 2022).

#### Mental Health Continuum-Short Form (MHC-SF; Lamers et al., 2011)

The MHC-SF is a commonly used and well-validated measure to assess well-being. The short version consists of 14 items that assess experiences over the past month, using a 6-point Likert scale from 0 (*Never*) to 5 (*Everyday*). There are three subscales that each assess Emotional, Social and Psychological Well-Being. Items from each subscale were summed, with higher scores indicating greater well-being. The MHC-SF subscales demonstrated excellent reliability (Cronbach’s α = .84 to .92), comparable to previous studies (Cronbach’s α = .91 to .97 overall, Higgins et al., 2021; Norén et al., 2023; Subramaniam et al., 2020).

#### Experience Questionnaire (EQ; Fresco et al., 2007)

The EQ is a validated 20-item measure designed to assess decentering and rumination. Only the 11-item decentering subscale was administered in this study. Participants indicated how often each statement applied to them in the past month, on a 5-point Likert scale from 1 (*Never*) to 5 (*All the Time*), with higher scores indicating higher levels of decentering. The EQ demonstrated excellent reliability (Cronbach’s α = .90 to .94), comparable to previous studies (Cronbach’s α = .83 to .90, Fresco et al., 2007).

#### List of Threatening Experiences Questionnaire (LTE; Brugha et al., 1985)

The LTE is a 12-item self-report measure where participants indicate whether they have experienced various stressful events (e.g., physical illness/serious injury, death, marital separation) in the past month (yes or no). The current study modified the measure to better capture change over time by indicating whether it is new or ongoing event. The physical illness/injury item (new or ongoing) was used as a covariate in all models to account for physical conditions that are not the focus of the current study but may impair functioning (e.g., mobility, self-care), given that the WHODAS 2.0 asks about both physical and mental conditions.

### DATA ANALYSIS

In Mplus 8.11 (Muthén & Muthén, 1998), multi-level structural equation modelling (MSEM) was used to model the two-level nested data structure, with survey responses (*N* = 12) nested within participants (*N* = 386). Thus, analyses model fluctuations within an individual (within-person) and individual differences over the course of the year (between-person). Within MSEM, within-person variables are implicitly person-centered, and the within- and between-person variance are completely distinct (i.e., orthogonal; Sadikaj et al., 2021). Intraclass correlations (ICCs) indicate the proportion of variability attributed to individual differences in each variable (with the remaining proportion attributable to within-person variance and error). Within-person models were specified at the within-person level, with unrestricted variances and covariances at the between-person level.

Across all models, the following variables were modelled as latent and tested with confirmatory factor analysis (CFA): *Symptoms* were defined by four IDAS subscales, *Impairment* was defined by six WHDOAS subscales, and *Well-Being* was defined by three MHC-SF subscales. Robust maximum likelihood estimation (MLR) was used for CFA, and good model fit was defined as a root mean square error of approximation (RMSEA) around .100 or smaller, comparative fit index (CFI) greater than .90 to .95, and standardised root mean square error residual (SRMR) values close to .08 or below (Brown, 2015). R^2^ was also consulted where available to reflect variance accounted for by the predictors, with .01 considered small, .06 medium, and .13 large for within-person effects (Cohen et al., 2003; Fairchild et al., 2009).

MSEM was conducted using Bayesian estimation, which was advantageous as it accounts for missing data and nonnormal distributions without biasing parameter estimates, as well as appropriately models asymmetric credibility intervals of indirect effects (which is currently not possible with MLR estimators in Mplus). Effects are interpreted as significant if the 95% credibility interval does not contain zero (Muthén & Asparouhov, 2012). The primary analysis examined moderated mediation at the within-person level with all measurements at the same timepoint using a two-staged approach. First, a simple mediation model was tested with latent variables (symptoms to impairment to well-being). Second, Decentering (specified as an observed variable) was added as a moderator of the symptom to impairment path, and a moderator of the impairment to well-being path (see Figure 1 for an illustration). Physical illness/injury was included as a covariate for Impairment and Well-Being in both models. Latent moderated SEM (Klein & Moosbrugger, 2000) was used to specify interactions between Symptoms and Decentering, and Impairment and Decentering (declaring variables as within-person in Mplus). Significant interaction effects were probed using the regions of significance approach facilitated by Preacher et al.’s (2006) calculator. This approach depicts the magnitude, direction, and significance of the association of the independent and dependent variables at all values of the moderator.

The exploratory model examined the same moderated mediation model described above, on the between-person level. However, this model did not converge upon a proper solution, despite several attempts to troubleshoot. Therefore, we instead estimated within- and between-person factor scores for symptoms, impairment, and decentering, and then saved the between-person factor scores for use in the between-person moderated mediation model.

Secondary analyses examined the mediation model with each variable temporally separated by one month and modelled as latent variables. As temporal associations were of interest, moderation was not as relevant in the secondary analyses, and the models were already quite computationally complex. Furthermore, given the quick timescale of decentering’s effects, decentering for 1 month seemed unlikely to impact experience and functioning 1 month later or more. Therefore, decentering was not included in the prospective model. Lagged variables were created to reflect responses to each measure at the prior timepoint. One model was analysed with Symptoms at T1 as the independent variable, Impairment at T2 as the mediator, and Well-Being at T3 as the dependent variable, consistent with the hypothesised direction of effects. Another model was analysed in the reverse-order, with Well-Being at T1, Impairment at T2, and Symptoms at T3. Both models controlled for the effects of physical illness/injury at T2 on Impairment and the outcome.

## Results

### SUMMARY STATISTICS AND PRELIMINARY ANALYSES

Descriptive statistics, ICCs and within-person correlations among each variable are presented in Table 1. The between-person descriptives and correlation matrix is presented in the online supplement (Supplementary Table 1). All ICCs were greater than .70, indicating that most of the variance was due to individual differences across the study, whereas less than 30% of the variance was due to fluctuations over time within-persons (and error variance). Thus, there was substantial stability over time, but adequate change to examine within-person associations. All variables were significantly correlated in the expected directions. Symptoms, well-being, and impairment in a given month all had small to moderate associations, and Dysphoria and Anxious Mood tended to be more strongly correlated with impairment domains compared to other symptoms. Within each month, greater decentering was moderately associated with greater well-being and with lower symptoms and impairment.

The results of the within-person 3-factor confirmatory factor analysis for symptoms, well-being, and impairment scales within a given month are presented in Supplementary Table 2. Fit was acceptable (RMSEA = .057, CFI = .952, SRMR = .051), and no modifications were made. Factor loadings ranged from .49 to .90 for Symptoms, .61 to .75 for Impairment, and .75 to .86 for Well-Being. Factor correlations were significant and moderate in size (*r* = .63 for Symptoms and Impairment, *r* = −.50 for Symptoms and Well-Being, *r* = −.40 for Impairment and Well-Being). For the exploratory between-person model using factor scores for each scale, fit was also acceptable (RMSEA = .043, CFI = .932, SRMR = .049), and no modifications were made (see Supplementary Table 3 for factor loadings).

### PRIMARY ANALYSIS: MODERATED-MEDIATION

The results of the simple mediation and moderated mediation models are provided in Figure 1, modelling concurrent associations within a given month. In the simple mediation model (Figure 1A), all direct paths and the indirect effect were statistically significant in expected directions. This suggests that Impairment partially explains the inverse association between Symptoms and Well-Being within a given month, accounting for 26% of the variance in Well-Being. Next, we tested moderated mediation by adding Decentering as a moderator of the *a* and *b* paths in the mediation model (Figure 1B). The direct effect between Symptoms and Well-Being was significant in the expected direction, as well as the path between Symptoms and Impairment. The interaction effect between Symptoms and Decentering was negative and statistically significant. However, the path between Impairment and Well-Being, the indirect effect, and the interaction between Impairment and Decentering were not significant.

The significant interaction between Symptoms and Decentering was further probed, revealing that the positive association between Symptoms and Impairment weakened as Decentering increased, though it remained significant at all levels of Decentering (see Figure 2). This suggests that on any given month, a greater tendency to decenter (relative to one’s norm) reduced the strength of the association between Symptoms and Impairment. However, in contrast to the simple mediation model, the association between Symptoms and Well-Being was not mediated by Impairment when Decentering was included.

The exploratory between-person model is presented in Supplementary Figure 1. The results were similar to that of the within-person model described above, in that Impairment did not mediate the association between Symptoms and Well-Being, and there was an interaction between Symptoms and Decentering in predicting Impairment (but not between Impairment and Decentering in predicting Well-Being). The only difference was that the direct effect between Symptoms and Well-Being was not significant at the between-person level. Regions of significance analysis of the Symptoms x Decentering interaction (Supplementary Figure 2) indicated that the positive association between Symptoms and Impairment weakened as Decentering increased, though it remained significant at all levels of Decentering.

### SECONDARY ANALYSES: MEDIATION OVER TIME

The results of the temporally-separated prospective mediation models are provided in Figure 3, with Figure 3A showing Symptoms predicting later Well-Being via Impairment (labelled “main model”) and Figure 3B showing Well-Being predicting later Symptoms via Impairment (labelled “reverse-ordered model”). Both models explained small amounts of variance in the outcome (*R*^2^ = .033 in the main model, and .022 in the reverse-ordered model). Results of the main model were consistent with the concurrent primary mediation model. The direct path between Symptoms at T1 and Well-Being at T3 was significant, as well as paths between Symptoms and Impairment, and Impairment and Well-Being. The indirect effect was also significant. In the reverse-ordered model, Well-Being at T1 was associated with less impaired function at T2, which was associated with fewer symptoms at T3, and the indirect effect was significant (Figure 3B). However, the direct effect between Well-Being at T1 and Symptoms at T3 was not significant. Therefore, Impairment partially accounted for the effects of Symptoms on subsequent Well-Being and fully accounted for the effects of Well-Being on subsequent Symptoms. As both models showed a significant indirect effect, bidirectional mediation was observed. Notably, the reverse-ordered model showed a descriptively larger indirect effect than the main model, with nonoverlapping 95% CIs.

## Discussion

The present study examined the role of impairment in explaining the association between symptoms and well-being over time, and the role of decentering as a protective factor. Results revealed two novel findings. First, decentering reduced the impact of one’s symptoms on impairment on a given month, but not the impact of impairment on well-being. This was also observed at the between-person level, suggesting individual differences in decentering reduce the association of symptoms and impairment. Second, prospective analyses revealed that the mediation effects of impairment were bidirectional. That is, impairment accounted for symptom’s later impact on well-being, and for well-being’s later impact on symptoms. These findings extend upon previous studies as we examined fluctuations over time, providing novel insight into potential causal associations that can inform interventions.

### PROTECTIVE ROLE OF DECENTERING

Results showed that in months where symptoms were elevated relative to one’s norm, well-being was negatively impacted via increases in impairment. There was a significant interaction between symptoms and decentering, consistent with our hypotheses, suggesting that when a person is able to decenter from distress, symptoms had less of an impact on functioning at that time (and overall throughout the study). This finding is a novel contribution to the literature, as reasons for the variation in the symptom-impairment link were relatively unknown. This result suggests that the way in which a person copes with or responds to their symptoms explains some variation in their impairment levels. Specifically, viewing distressing thoughts/feelings objectively and separately from oneself can reduce the detrimental effects of symptoms on day-to-day functioning and thus allow one to function more adequately. Conversely, on months when decentering is lower, or for people who are less able to decenter, symptoms were more strongly linked to impairments in functioning. Given prior literature about state decentering, it is possible that this reflects buffering effects of momentary elevations in decentering (Naragon-Gainey et al., 2023).

We also found that general tendencies to decenter interrupted the link between overall symptoms and impairment throughout the year (i.e., between-person effects). Note that the analyses allowed for a separation of within-person and between-person effects, so moderation findings across both levels suggest both concurrent, time-linked impacts of greater decentering, and individual differences for people who generally decenter more. While state and trait effects may interact in complex ways not modelled here, these results provide initial support that both timescales may be important. Future studies should also examine possible confounding individual differences (e.g., traits such as emotional stability/low neuroticism) to better understand the specificity of decentering in the between-person results.

Turning to the rest of the moderated mediation model, greater decentering was also directly related to greater well-being that month. Impairment, however, was not significantly related to well-being, and decentering did not moderate this relationship, contrary to hypotheses. Moreover, although symptoms directly predicted well-being that month, impairment no longer played a mediating role when decentering was considered. In other words, impairment does not predict or explain well-being when decentering is accounted for. One explanation for this could be that the moderated-mediation model was more complex and reduced statistical power, making it harder to detect smaller effects. It is also possible that the addition of decentering alters the whole network of associations in complex ways that were not fully modelled or understood currently. Statistically, potential overlapping variance and suppression effects way have occurred because after accounting for the symptom-decentering interaction, the unique residual variance in impairment was reduced, and so may have little predictive value for well-being. Conceptually, this suggests that mediation may be conditional and therefore may not be robust across levels of decentering. For example, perhaps when decentering is low and symptoms and impairment are strongly linked, then impairment more greatly impacts well-being. Conversely, when decentering is high and the symptom-impairment link is reduced, impairment is lessened and may no longer uniquely predict well-being or is less relevant.

### PROSPECTIVE TEMPORAL ASSOCIATIONS

We also examined indirect effects without considering levels of decentering. Impairment was a significant mediator of symptom-well-being associations when examined in three ways: concurrently within a given month, prospectively from prior symptoms to later well-being, and prospectively from prior well-being to later symptoms. This pattern of findings highlights the immediate effects of these variables, as well as the potential cumulation of impairment over time on subsequent well-being and symptoms. That is, prolonged symptoms have long term impacts on functioning, and prolonged impairment reduces well-being. A feedback loop was further demonstrated, whereby one’s well-being also predicted later symptoms, via effects on impairment.

While prior research had established a bidirectional association between symptoms and well-being (Lamers et al., 2015; Joshanloo & Blasco-Belled, 2023), it was not known *how* they influenced one another. Our finding that impairment contributes to this bidirectional association underlines the importance of one’s functioning in understanding how symptoms and well-being are interconnected, as they mutually reinforce one another. As such, the persistence of impairment over time increases risk for both lowered well-being and elevated symptoms, which also impact one another. In this way, impairment may contribute to the chronicity, maintenance and severity of the symptom-low well-being cycle over time (Moorey, 2010). Targeting impairment as an underlying mechanism of this cycle could thus disrupt symptom maintenance and facilitate recovery as defined by the Recovery Paradigm (i.e., resilience/increased well-being in addition to symptom reduction, Jacob, 2015). Decentering was shown to mitigate the effect of symptoms on impairment in the short-term, but it is unclear how decentering, or other factors, could prevent this cycle long-term. This would be an important avenue for future research given that greater use of psychological strengths over time is predictive of positive changes in well-being (Wood et al., 2011).

### CLINICAL IMPLICATIONS

Decentering is a malleable skill included in many psychological treatments (e.g., mindfulness therapies, such as Acceptance Commitment Therapy and Mindfulness-Based Cognitive Therapy), with an aim of reducing the persistence and exacerbation of distress via rumination or other processes. The current study highlights decentering as a potential way to also prevent impairment in the moment, which is regarded as an important outcome in treatment settings. Specifically, teaching decentering in therapy may facilitate higher levels of functioning in important life domains, particularly in times of elevated symptoms. Consistently responding to one’s symptoms with a decentered approach may promote resilience to the effects of symptoms, as longitudinal results suggest that over time, these constructs are interrelated via impairment. Practicing decentering skills that preserve functioning under distress may help to facilitate other therapy components such as behavioural activation and values-focused treatments, as it allows for flexibility in choosing behaviours that align with personal values (Mazzucchelli et al., 2010; Stenhoff et al., 2020). Moreover, given the reinforcing spiral, clinicians could treat the person’s primary concerns (elevated symptoms, impairment, or low well-being) and see flow on effects on the other, with the core mechanism being impairment levels. This study adds to growing literature to suggest that in addition to symptom reduction, functional improvement is a key therapeutic outcome that should be emphasised for prevention and treatment of low well-being and symptoms.

### LIMITATIONS AND FUTURE DIRECTIONS

Future studies can build upon the present study by addressing several limitations. Regarding the prospective models, this study did not assess moderation effects long term. More specifically, it was difficult to establish that current decentering would impact the associations a month later, so shorter-term effects (i.e., concurrent) were deemed most relevant for moderation. Thus, to capture prospective effects of decentering, it is recommended to test moderating effects at shorter time-frames (e.g., hours or days) particularly as functioning difficulties may be more apparent at certain tasks or times of day. Longer term, future studies could explore a wider range of protective factors (e.g., other psychological strengths, coping skills, physical health) at various stages of the mediation model and see how they interact and alter changes in symptoms, impairment and well-being over time. It would also be valuable to assess moderation in the reverse-ordered model to prevent elevations in symptoms.

Next, even though one quarter of participants in the present study met criteria for an internalising disorder, future studies should explore these research questions in clinical samples where symptoms may be more extreme and impairment more pervasive or difficult to prevent. Moreover, subjective impairment ratings are meaningful in that they capture perceived disability, but as they can be quite subjective, clinician-rated impairment may be valuable to reduce potential self-report biases, using clinical-judgement to assess norms vs. impaired functioning. Finally, although 53.4% of the sample reported experience with meditation practice, there was a wide range of experience (0 to 52 years), and meditation was not formally taught. This naturalistic variation in meditation practice may be informative about real-world variation in decentering practice and processes. Future studies may consider the effects of formal meditation training to elicit decentering and examine the effects of this, such as through intervention or experimental design.

### CONCLUSION

By examining several mental health constructs over the course of a year, this study provided valuable insights into the relationships between one’s internalising symptoms, well-being, and functioning. Decentering was supported as a protective factor that reduces the link between elevated symptoms and impairment, but not impairment and well-being. Further research is needed to uncover different ways to protect well-being in addition to this, particularly because low well-being also predicted elevated symptoms via impairment over time.

## Supplementary Material

Supplement

Supplementary data to this article can be found online at https://doi.org/10.1016/j.beth.2026.04.002.

## Figures and Tables

**FIGURE 1:**
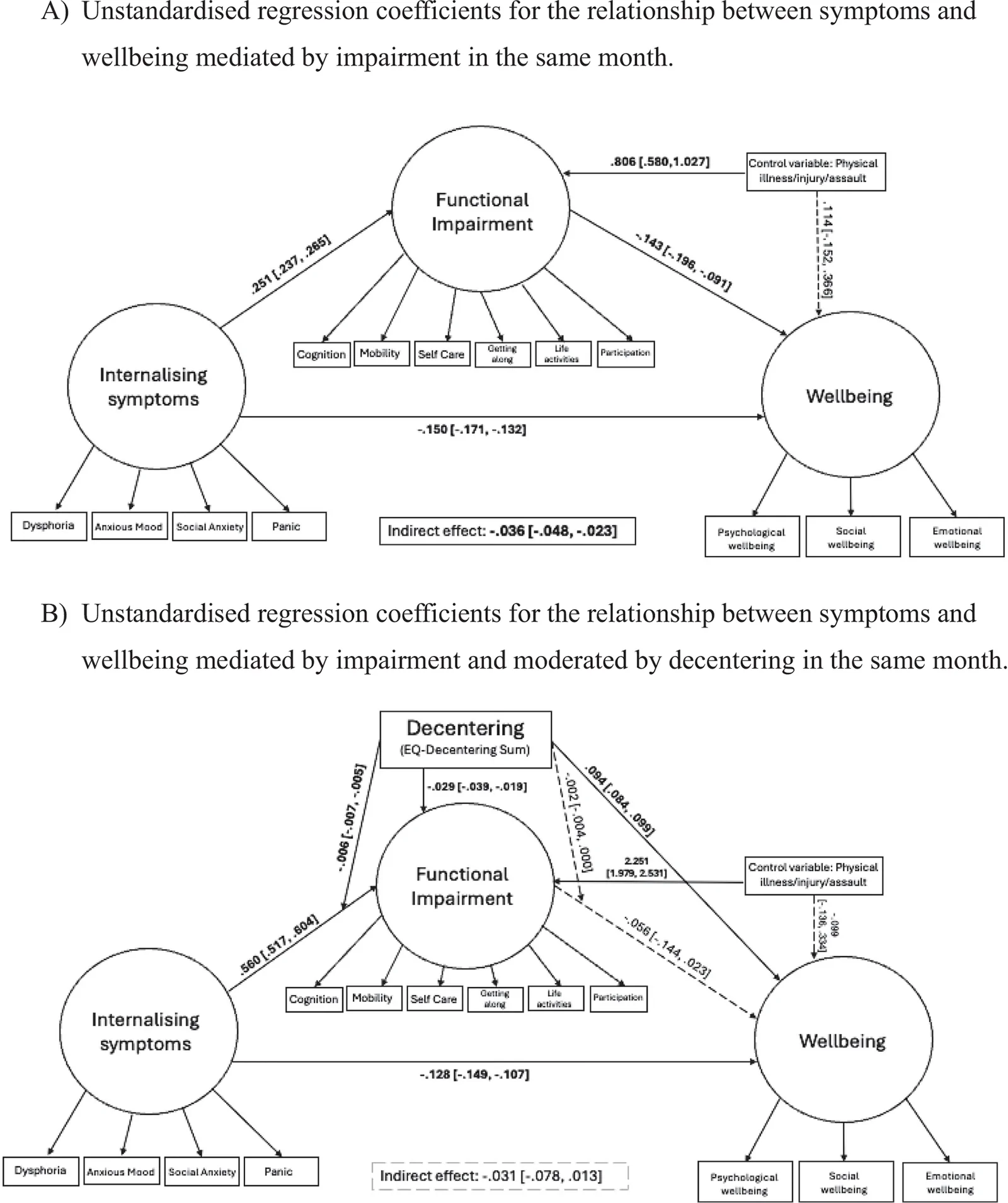
Primary Analysis: within-person mediation and moderated-mediation model. (A) Unstandardised regression coefficients for the relationship between symptoms and well-being mediated by impairment in the same month. Note. Values in parentheses = 95% confidence intervals. Bolded = significant paths, Dashed = non-significant paths. EQ-Decentering = Experience Questionnaire Decentering subscale.

**FIGURE 2:**
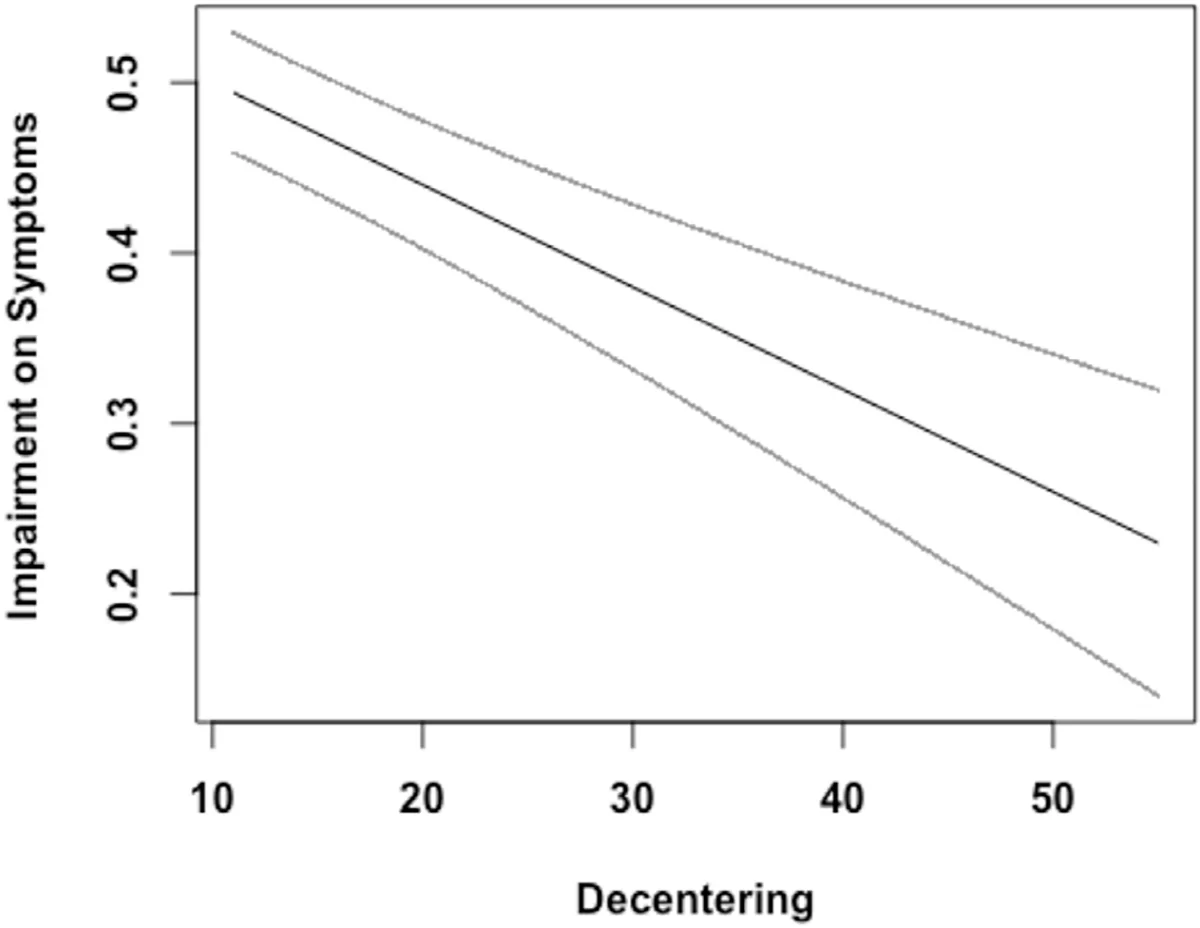
Plot of significant within-person interaction between Symptoms and Decentering predicting Impairment. Note. The two curved lines around the slope are confidence bands, reflecting the range of values where the effect is statistically significant at *p* < .05 (i.e., where the confidence bands do not include zero).

**FIGURE 3:**
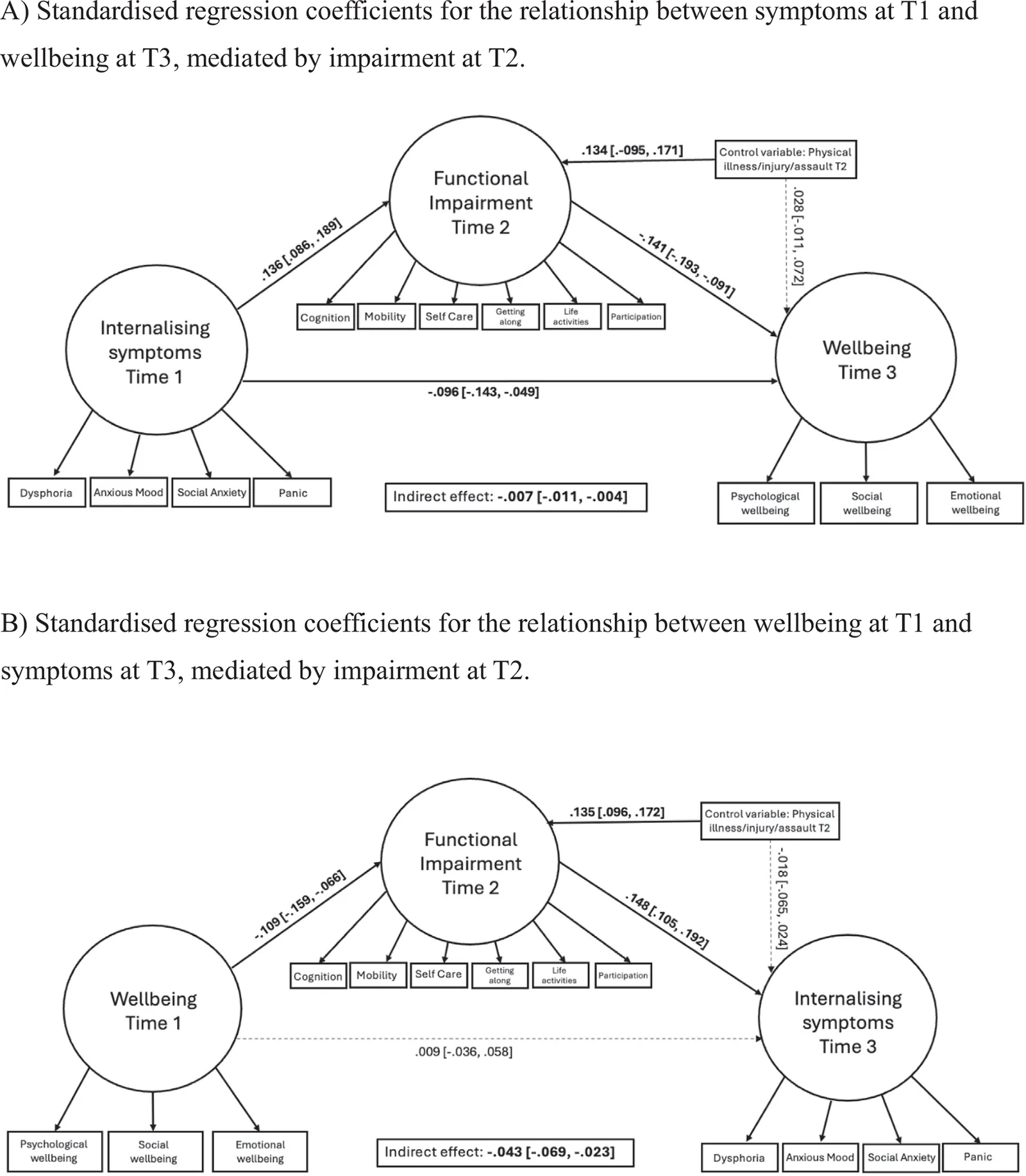
Secondary Analyses: Temporally separated within-person mediation models. (A) Standardised regression coefficients for the relationship between symptoms at T1 and well-being at T3, mediated by impairment at T2. (B) Standardised regression coefficients for the relationship between well-being at T1 and symptoms at T3, mediated by impairment at T2. Note. Values in parentheses = 95% confidence intervals. Bolded = significant paths, Dashed = non-significant paths. Each timepoint is separated by approximately 1 month.

**Table 1:** Descriptive statistics, intraclass correlations, and within-person correlation matrix for all subscales.

		SD_W_	Range	ICC	1	2	3	4	5	6	7	8	9	10	11	12	13	14

1.	Panic (IDAS-II)	2.63	8–40	.707	–	.40	.43	.42	.28	.26	.26	.26	.28	.33	−.24	−.16	−.19	−.19
2.	Social Anxiety (IDAS-II)	2.26	5–25	.752		–	.42	.36	.31	.16	.19	.34	.22	.26	−.22	−.16	−.16	−.19
3.	Dysphoria (IDAS-II)	4.50	9–45	.685			–	.75	.44	.27	.28	.39	.43	.44	−.45	−.30	−.36	−.36
4.	Anxious Mood (IDAS-II)	4.11	7–35	.666				–	.36	.22	.23	.31	.35	.38	−.40	−.27	−.30	−.32
5.	Cognition (WHODAS 2.0)	2.19	6–30	.721					–	.47	.46	.58	.49	.47	−.27	−.19	−.25	−.22
6.	Mobility (WHODAS 2.0)	1.67	5–25	.747						–	.53	.40	.42	.45	−.18	−.12	−.15	−.15
7.	Self-Care (WHODAS 2.0)	1.28	4–20	.697							–	.42	.37	.40	−.19	−.11	−.15	−.17
8.	Getting Along (WHODAS 2.0)	2.05	5–25	.708								–	.37	.47	−.28	−.19	−.25	−.23
9.	Life Activities (WHODAS 2.0)	3.76	8–40	.716									–	.57	−.29	−.20	−.26	−.23
10.	Participation (WHODAS 2.0)	2.78	8–40	.765										–	−.32	−.21	−.27	−.28
11.	Emotional Well-Being (MHC-SF)	1.96	0–15	.704											–	.57	.66	.32
12.	Social Well-Being (MHC-SF)	2.96	0–25	.760												–	.67	.28
13.	Psychological Well-Being (MHC-SF)	3.46	0–30	.746													–	.35
14.	Decentering (EQ)	4.65	11–55	.699														–

Note. W = within persons. IDAS-II = Inventory of Depression and Anxiety Symptoms – Expanded version. WHODAS 2.0 = World Health Organisation Disability Assessment Schedule 2.0. MHC-SF = Mental Health Continuum-Short Form. EQ = Experience Questionnaire.

The within-person means are set to zero by default in the model. All correlations were statistically significant at *p* < .001. Range = possible range.
