# Supplementary material for: The Protective Effect of Decentering on the Links Between Internalising Symptoms, Functional Impairment and Wellbeing
Source: Behav Ther. Author manuscript; Available in PMC 2026 Nov 11. (PMC13754401; doi:10.1016/j.beth.2026.04.002)
Supplement: Supplementary file 1 [file NIHMS2219096-supplement-Supplement.doc]

**Supplementary Table 1**

Between-Person Descriptive Statistics.

|  | Mean | SD | 1 | 2 | 3 | 4 | 5 | 6 | 7 | 8 | 9 | 10 | 11 | 12 | 13 | 14 |
| --- | --- | --- | --- | --- | --- | --- | --- | --- | --- | --- | --- | --- | --- | --- | --- | --- |
| 1. Panic (IDAS-II) | 11.85 | 4.07 | - |  |  |  |  |  |  |  |  |  |  |  |  |  |
| 2. Social Anxiety (IDAS-II) | 9.73 | 3.93 | .74 | - |  |  |  |  |  |  |  |  |  |  |  |  |
| 3. Dysphoria (IDAS-II) | 20.07 | 6.63 | .76 | .81 | - |  |  |  |  |  |  |  |  |  |  |  |
| 4. Anxious Mood (IDAS-II) | 17.51 | 5.80 | .73 | .77 | .91 | - |  |  |  |  |  |  |  |  |  |  |
| 5. Cognition (WHODAS 2.0) | 10.35 | 3.52 | .76 | .79 | .84 | .74 | - |  |  |  |  |  |  |  |  |  |
| 6. Mobility (WHODAS 2.0) | 7.30 | 2.87 | .60 | .51 | .52 | .47 | .70 | - |  |  |  |  |  |  |  |  |
| 7. Self-Care (WHODAS 2.0) | 5.54 | 1.94 | .66 | .60 | .67 | .57 | .78 | .77 | - |  |  |  |  |  |  |  |
| 8. Getting Along (WHODAS 2.0) | 8.98 | 3.19 | .68 | .78 | .77 | .68 | .82 | .64 | .69 | - |  |  |  |  |  |  |
| 9. Life Activities (WHODAS 2.0) | 15.62 | 5.97 | .70 | .62 | .78 | .68 | .88 | .74 | .76 | .73 | - |  |  |  |  |  |
| 10. Participation (WHODAS 2.0) | 13.57 | 5.01 | .72 | .65 | .76 | .69 | .81 | .74 | .75 | .82 | .82 | - |  |  |  |  |
| 11. Emotional Wellbeing (MHC-SF) | 8.93 | 3.02 | -.47 | -.58 | -.70 | -.62 | -.59 | -.43 | -.45 | -.62 | -.52 | -.53 | - |  |  |  |
| 12. Social Wellbeing (MHC-SF) | 11.58 | 5.27 | -.42 | -.52 | -.58 | -.54 | -.50 | -.40 | -.39 | -.55 | -.42 | -.45 | .86 | - |  |  |
| 13. Psychological Wellbeing (MHC-SF) | 17.17 | 5.76 | -.45 | -.59 | -.68 | -.58 | -.59 | -.43 | -.45 | -.60 | -.52 | -.49 | .94 | .90 | - |  |
| 14. Decentering (EQ) | 35.19 | 7.08 | -.47 | -.61 | -.69 | -.68 | -.57 | -.34 | -.40 | -.53 | -.51 | -.46 | .72 | .70 | .78 | - |

*Note.* All correlations were statistically significant at *p* < .001. IDAS-II = Inventory of Depression and Anxiety Symptoms – Expanded version. WHODAS 2.0 = World Health Organisation Disability Assessment Schedule 2.0. MHC-SF = Mental Health Continuum-Short Form. EQ = Experience Questionnaire.

**Supplementary Table 2**

Confirmatory Factor Analysis for Within-Person Latent Variables: Factor Loadings and Correlations.

| **Model** |  | Factors |  |  |
| --- | --- | --- | --- | --- |
|  | Symptoms | Impairment | Wellbeing |  |
| **Primary Analysis** | | | |  |
| *Factor Loadings* |  |  |  |  |
| IDAS Dysphoria | .902 |  |  |  |
| IDAS Anxious Mood | .815 |  |  |  |
| IDAS Social Anxiety | .485 |  |  |  |
| IDAS Panic | .513 |  |  |  |
| WHODAS Cognition |  | .749 |  |  |
| WHODAS Mobility |  | .630 |  |  |
| WHODAS Self Care |  | .606 |  |  |
| WHODAS Get Along |  | .677 |  |  |
| WHODAS Life activities |  | .674 |  |  |
| WHODAS Participation |  | .715 |  |  |
| MHC-SF Emotional |  |  | .779 |  |
| MHC-SF Social |  |  | .752 |  |
| MHC-SF Psychological |  |  | .860 |  |
| *Factor Correlations* |  |  |  |  |
| Symptoms | - |  |  |  |
| Impairment | .626 | - |  |  |
| Wellbeing | -.496 | -.404 | - |  |

*Note.* All estimates are standardised and statistically significant (p < .05). IDAS-II = Inventory of Depression and Anxiety Symptoms – Expanded version. WHODAS 2.0 = World Health Organisation Disability Assessment Schedule 2.0. MHC-SF = Mental Health Continuum-Short Form.

**Supplementary Table 3**

Confirmatory Factor Analysis for Between-Person Latent Variables: Factor Loadings and Correlations.

| **Model** |  | Factors |  |  |
| --- | --- | --- | --- | --- |
|  | Symptoms | Impairment | Wellbeing |  |
| **Primary Analysis** | | | |  |
| *Factor Loadings* |  |  |  |  |
| IDAS Dysphoria | .979 |  |  |  |
| IDAS Anxious Mood | .926 |  |  |  |
| IDAS Social Anxiety | .855 |  |  |  |
| IDAS Panic | .806 |  |  |  |
| WHODAS Cognition |  | .955 |  |  |
| WHODAS Mobility |  | .775 |  |  |
| WHODAS Self Care |  | .845 |  |  |
| WHODAS Get Along |  | .870 |  |  |
| WHODAS Life activities |  | .923 |  |  |
| WHODAS Participation |  | .897 |  |  |
| MHC-SF Emotional |  |  | .954 |  |
| MHC-SF Social |  |  | .910 |  |
| MHC-SF Psychological |  |  | .987 |  |
| *Factor Correlations* |  |  |  |  |
| Symptoms | - |  |  |  |
| Impairment | .885 | - |  |  |
| Wellbeing | -.697 | -.610 | - |  |

*Note.* All estimates are standardised and statistically significant (p < .05). IDAS-II = Inventory of Depression and Anxiety Symptoms – Expanded version. WHODAS 2.0 = World Health Organisation Disability Assessment Schedule 2.0. MHC-SF = Mental Health Continuum-Short Form.

**Supplementary Figure 1**

Exploratory Analysis: Between-Person Moderated-Mediation Model.

Unstandardised Regression Coefficients for the Relationship Between Symptoms and Wellbeing Mediated by Impairment and Moderated by Decentering.

*Note.* Values in parentheses = 95% confidence intervals. Bolded = significant paths, Dashed = non-significant paths. EQ-Decentering = Experience Questionnaire Decentering subscale.

**Supplementary Figure 2**

Plot of Significant Between-Person Interaction Between Symptoms and Decentering Predicting Impairment.

*Note.* The two curved lines around the slope are confidence bands, reflecting the range of values where the effect is statistically significant at *p* < .05 (i.e., where the confidence bands do not include zero).
